# Association of the 894G>T polymorphism in the endothelial nitric oxide synthase gene with risk of acute myocardial infarction

**DOI:** 10.1186/1471-2350-9-43

**Published:** 2008-05-21

**Authors:** George K Andrikopoulos, Dimitris K Grammatopoulos, Stylianos E Tzeis, Sevasti I Zervou, Dimitris J Richter, Michalis N Zairis, Elias J Gialafos, Dimitris C Sakellariou, Stefanos G Foussas, Antonis S Manolis, Christodoulos I Stefanadis, Pavlos K Toutouzas, Edward W Hillhouse, The GEMIG study investigators

**Affiliations:** 1 1st Cardiac Department, Evaggelismos Hospital, Athens, Greece; 2 Department of Biological Sciences, University of Warwick Medical School, Warwick, UK; 3 Department of Biological Sciences, University of Warwick, Warwick, UK; 4 Cardiac Department Euroclinic Hospital, Athens, Greece; 5 Cardiac Department Tzaneion Hospital, Piraeus, Greece; 6 Cardiac Department, Laikon Hospital, Athens, Greece; 7 1st Cardiac dpt., University of Athens Medical School, Greece; 8 Director of Hellenic Heart Foundation, Athens, Greece; 9 Dean of School of Medicine, University of Leeds, Leeds, UK

## Abstract

**Background:**

This study was designed to investigate the association of the 894G>T polymorphism in the eNOS gene with risk of acute myocardial infarction (AMI), extent of coronary artery disease (CAD) on coronary angiography, and in-hospital mortality after AMI.

**Methods:**

We studied 1602 consecutive patients who were enrolled in the GEMIG study. The control group was comprised by 727 individuals, who were randomly selected from the general adult population.

**Results:**

The prevalence of the Asp298 variant of eNOS was not found to be significantly and independently associated with risk of AMI (RR = 1.08, 95%CI = 0.77–1.51, P = 0.663), extent of CAD on angiography (OR = 1.18, 95%CI = 0.63–2.23, P = 0.605) and in-hospital mortality (RR = 1.08, 95%CI = 0.29–4.04, P = 0.908).

**Conclusion:**

In contrast to previous reports, homozygosity for the Asp298 variant of the 894G>T polymorphism in the eNOS gene was not found to be associated with risk of AMI, extent of CAD and in-hospital mortality after AMI

## Background

A single base exchange (G^894^→T) in exon 7 of the human endothelial nitric oxide synthase (eNOS) gene results in a Glu→Asp substitution at residue 298 of the eNOS gene. The functional significance of this single nucleotide polymorphism remains an issue of controversy since homozygosity for the Asp298 variant has been related to reduced enzyme activity [1] and basal NO production [2], possibly due to increased susceptibility to proteolytic cleavage [3], although more recent reports have convincingly demonstrated that this preferential cleavage could be a methodological artifact [4,5].

In accordance to the hypothesis that this polymorphism may have an unfavorable effect on NO bioavailability, homozygosity for the Asp298 variant has been reported to influence vascular coronary reactivity [6], responsiveness to a-adrenergic stimulation [7], and event free survival in patients with nonischemic cardiomyopathy [8]. Based on these reports a number of association studies have positively associated the presence of the Asp298 variant with risk of AMI [9,10], carotid atherosclerosis [11], early atherogenesis [12], and coronary in-stent restenosis [13], while several studies have found no evidence for an association between the 894G>T polymorphism and premature CAD [14-17].

Based on data derived from a multicentre genetic epidemiological study, we examined the association of this genetic variant of eNOS gene with risk of AMI in a relatively homogeneous, in terms of ethnic and cultural background, low coronary risk, Caucasian population.

## Methods

### Patient population

The GEMIG study (Genetics and Epidemiology of acute Myocardial Infarction in the Greek population) is a multicenter study designed to evaluate the genetic predisposition of AMI and prognosis after AMI in the Greek population. A total of 1602 consecutive patients, admitted in hospital with the diagnosis of AMI, who have been successfully genotyped for the 894G>T polymorphism were enrolled in the study. The control group consisted of 805 adults (aged > 30 years old) who were randomly selected from the city records. Blood samples for genetic analyses were obtained from 794 of the study participants. Thirty-two out of the 794 eligible subjects had clinical or electrocardiographic evidence of a possible MI and were not included in the control group. Out of the remaining 762 subjects, 727 were successfully genotyped for the 894G>T polymorphism. The study methodology, participating centers, study investigators and findings have been published in details elsewhere [18-20]. The scientific committee of the study and the local ethics committees of all participating institutes approved the study protocol. Prior to that the scientific and ethics committee of the coordinating center (Cardiac department of Athens University, Hippokration Hospital, Athens, Greece) had approved the study protocol. The protocol of the study affected neither the diagnostic procedures, nor the therapeutic interventions applied to the studied patients who gave informed consent for their participation.

### Special characteristics of the study population

The Greek population is a relatively homogeneous Caucasian population in terms of ethnic and cultural background, which presents low ischemic heart disease mortality rates and low incidence of CAD despite the relatively high prevalence of major coronary risk factors. The aforementioned characteristics have been verified in large scale epidemiological studies in the general population [21] and in patients with documented CAD [22].

### DNA analysis

In order to investigate the 894G>T polymorphism of eNOS gene, located in exon 7, oligonucleotide primers for polymerase chain reaction (PCR) were designed using the published sequence of the human eNOS (NOS 3) gene (Genbank/EMBL L10693–L10709) [23]. A coding sequence variant, a G→T substitution in exon 7 (at position 894) in codon 298, alters the amino acid at this residue from Glu to Asp. Genotyping of this polymorphism was performed by PCR amplification of exon 7, with the flanking intronic primers 5'-CAT-GAG-GCT-CAG-CCC-CAG-AAC-3' (sense) and 5'-AGT CAA-TCC-CTT-TGG-TGC-TCA-C-3' (antisense), followed by *Mbo*I restriction endonuclease digestion for 16 hours at 37°C. Non-denaturing, 3% agarose gel electrophoresis, was used to identify a single PCR product of 206 bp. Cleavage of the product into 119 bp and 87 bp fragments occurred in the presence of a T at nucleotide 894. The latter corresponds to Asp^298^, while no restriction digestion products were obtained in the absence of T at the same residue.

### Statistical analysis

Statistical analyses were performed with SPSS software (version 13.0, Chicago, IL, U.S.A.). A P value on a 2-sided test of 0.05 for group comparisons and 0.10 for interaction tests was considered statistically significant. Chi-square tests were used to compare genotype frequencies in different groups. Multivariate logistic regression analysis, with an allowance for age only, age and gender, or a group of the major cardiovascular risk factors (age, gender, hypercholesterolaemia, hypertension, diabetes mellitus, smoking and family history for CAD), was used to explore the impact of Asp298 variant on risk of AMI. Chi-square analysis was used to test the assumption of Hardy-Weinberg equilibrium.

## Results

**Figure 1:**
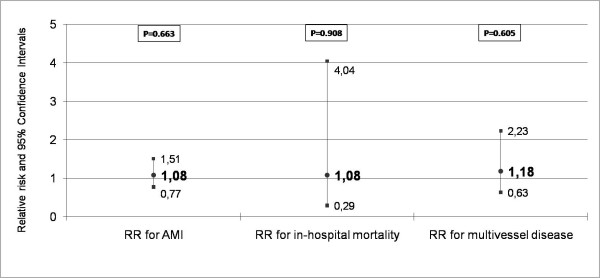
**Synopsis of the results of multivariate analyses.** The relative risks and the corresponding 95% confidence intervals for AMI, in-hospital mortality and multivessel disease in relation to the presence of the mutated (Asp/Asp) genotype are shown. Detailed description of the variables included in each multivariate analysis is presented in the results section of the manuscript.

The main baseline characteristics of the patients with AMI (cases) and of the subjects from the general population (controls) are presented in Table 1. The frequencies of the studied genotypes of the eNOS gene are shown in Figure 1. The observed frequencies of the studied alleles were in Hardy-Weinberg equilibrium in both cases (χ^2 ^= 0.20, P > 0.1) and controls (χ^2 ^= 1.65, P > 0.1).

**Table 1:** Baseline characteristics of the study population

	Cases* n = 1602	Controls† n = 727	P value
Age (years)	62 ± 13	58 ± 15	< 0.001
Male gender	79% (n = 1261)	43% (n = 314)	< 0.001
Diabetes	29% (n = 459)	11% (n = 77)	< 0.001
Cigarette smoking	64% (n = 1017)	33% (n = 242)	< 0.001
Hypercholesterolaemia	50% (n = 800)	33% (n = 241)	< 0.001
Hypertension	45% (n = 719)	32% (n = 230)	< 0.001
Heredity for CAD‡	26% (n = 412)	22% (n = 158)	0.040

*Cases = successfully genotyped patients with acute myocardial infarction

†Controls = successfully genotyped individuals from the general population without a history of myocardial infarction

‡CAD = coronary artery disease.

The frequency of the Asp/Asp genotype was not found to differ significantly between cases and controls in relation to the major coronary risk factors (gender, smoking status, diabetes mellitus, hypercholesterolemia, hypertension, obesity and family history of CAD). Furthermore, the average number of the aforementioned risk factors did not differ significantly in carriers versus non-carriers of the Asp/Asp genotype, in both cases (2.36 vs 2.32, P = 0.771) and controls (1.60 vs 1.81, P = 0.137).

### Homozygosity for the Asp allele and risk of AMI and in-hospital mortality of AMI

The frequency of the mutated genotype (Asp/Asp) did not differ significantly between cases and controls (11.2 vs 10.7%, P = NS). Similarly, the Glu/Asp (43.8 vs 40.9, P = NS) and the Glu/Glu (43.8 vs 40.9, P = NS) genotypes did not differ significantly between the study groups in univariate analysis. In backward stepwise logistic regression analysis with age, gender, smoking status, diabetes mellitus, hypercholesterolemia, hypertension, obesity and family history of CAD included as covariates, the presence of the Asp/Asp genotype was not found to be independently associated with AMI (RR = 1.08, 95%CI = 0.77–1.51, P = 0.663) (Figure 1).

Unadjusted in-hospital mortality of carriers and of non-carriers of the Asp/Asp genotype did not differ significantly (7.8 vs 7.7%, P = 0.939). Possession of this genotype was also not significantly associated with in-hospital mortality in multivariate analysis adjusted for age, gender, diabetes mellitus, hypertension, smoking status, obesity, anterior location of infarction, administration of thrombolysis, and echocardiographic left ventricular ejection fraction (RR = 1.08, 95%CI = 0.29–4.04, P = 0.908) (Figure 1).

### Homozygosity for the Asp298 allele in relation to the number of diseased vessels

Out of the studied AMI patients, 614 underwent coronary angiography during the hospitalization period. The frequencies of the Asp/Asp genotype in relation to the number of diseased vessels were 13.3, 8.1, and 9.3% in patients with one-, two-, and three-vessel disease, respectively (P = 0.192). In multivariate analysis adjusted for age, gender, diabetes mellitus, smoking status, hypertension, hypercholesterolemia, and obesity, the Asp/Asp variant was not found to be independently associated with the number of diseased vessels on coronary angiography (OR = 1.18, 95%CI = 0.63–2.23, P = 0.605) (Figure 1).

## Discussion

In contrast to previous reports, our results derived from a specifically designed, prospective, multicentre study do not support the hypothesis that homozygosity for the G^894^→T polymorphism, in eNOS gene is associated with increased risk of AMI. Furthermore, we found no relation of this polymorphism with angiographic extent of CAD and in-hospital mortality after AMI.

### Genetic association studies for ischemic heart disease and special characteristics of the present study

The extensive search for a genetic variant that would influence significantly coronary risk, independently of the major coronary risk factors, has been a target of cardiovascular research on genetic epidemiology during the last decades. However, genetic association studies for atherosclerotic cardiovascular diseases have resulted in conflicting, non-reproducible and rather disappointing results. Furthermore, publication bias has contributed to the contamination of literature with small association studies reporting positive results. Thus, data from association studies should be evaluated in the context of their major methodological limitations. The chance of a false linkage in association studies is high and the replication of findings, even in the same population, has been limited. In addition, highly selected or not representative groups of cases and controls, retrospective design, lack of multivariate analysis, and bias introduced by selection by death have also contributed to the plethora of contradictory results in the field [24,25]. In this study we enrolled a large number of consecutive patients hospitalized for AMI, aiming to avoid misclassification with subjects not really suffering from CAD. The control group was randomly selected from the general adult population included in the city catalogues aiming to avoid selection biases.

### The role of the 894G>T polymorphism on the function of eNOS

Endothelial NOS regulates NO synthesis by the endothelium which is demonstrated to exert a key role on coronary vasodilatation, vascular smooth muscle cell growth, endogenous antioxidant defense and platelet aggregation [26]. Several studies have examined whether the 894G>T polymorphism of the eNOS gene alters the functional profile of the protein, trying to provide a pathophysiological background which could support the clinical hypothesis of association between this polymorphism and several cardiovascular diseases. Initial studies demonstrated that the Asp298-encoded eNOS enzyme is more prone to proteolytic cleavage, resulting in reduced levels of functional eNOS and thus to a diminished steady-state eNOS activity [3]. However, it has been indicated that the reported preferential cleavage of the Asp298 eNOS variant is rather a methodological artifact attributed to nonspecific acid hydrolysis during sample preparation [3,4], while proper buffer selection and avoidance of acid conditions completely prevent Asp-eNOS proteolysis [5].

Furthermore, Golser et al. [27] reported that Asp298 variant purified from a yeast expression system does not influence enzyme function and can not explain endothelial dysfunction associated with this polymorphism, while Dosenko et al. [28] demonstrated that eNOS activity in isolated human platelets from 894T/T homozygotes was not significantly lower than in normal homozygotes. Further evidence supporting the lack of functional significance of this single nucleotide polymorphism was derived from the elegant study by McDonald et al. [5] who showed that Glu→Asp substitution at position 298 of the eNOS does not modulate either the subcellular localization and interaction with modulatory proteins of the enzyme or its activity in intact human endothelial cells. This is also consistent with the spatial location of the position 298 of the eNOS gene, which is situated externally, far from the binding sites of eNOS regulatory proteins [29,30], and thus this polymorphism is considered unlikely to alter the functional properties of the enzyme to a considerable extent. These data strongly doubt the functional significance of this polymorphism and favor the scenario that this eNOS variant might be an indirect marker of genetic association with other disease-related variants in either the eNOS gene or at other loci [5].

### Association of 894G>T polymorphism with risk of CAD

A metaanalysis of case-control studies evaluating the potential association between 894G>T eNOS polymorphism and the risk of CAD reached the conclusion that homozygosity for eNOS Asp298 allele was associated with a moderately, though significantly increased risk of CAD [OR = 1.31;95%CI = 1.13–1.51] [31]. The reported results are amenable to criticism due to the significant heterogeneity of the individual odds ratios incorporated in the calculation of the summary odds ratio. After excluding from the analysis the study with the most influential odds ratio, the authors abrogated the methodological limitation of heterogeneity, but the calculated risk of CAD was largely blunted and bordered on significance (summary OR = 1.17; 95%CI = 1.00–1.36; P = 0.05).

Antoniades et al. [10] in an elegantly designed study with 229 consecutive patients with premature AMI, demonstrated that homozygosity for this polymorphism is associated with a significantly increased risk for premature AMI. Although discordant at a first glance with our results, the conclusions of this study refer to a different, younger than our patient population, where the relative contribution of the 894G>T polymorphism to the susceptibility for AMI might be enhanced. Furthermore, the fairly large patient population of our study renders our results less prone to the caveat of sampling variability in case-control studies.

A large scale study on 5061 individuals of Japanese origin demonstrated no association of the 894G>T polymorphism with risk of AMI [15]. Spence et al. [16] using family-based association tests specifically designed for the study of the genetic basis of multifactorial diseases, found no evidence that the 894G>T eNOS gene polymorphism was related to the development of CAD in a total of 1023 Caucasian individuals.

In a recent study of 861 diabetic men no significant association was observed between 894G>T eNOS polymorphism and risk of CAD [17]. There is also discrepancy in the literature regarding the association between the 894G>T polymorphism and premature CAD. Granath and colleagues[14] studying 573 patients younger than 50 years reported the absence of association between 894G>T eNOS polymorphism and premature CAD, while in a recent smaller trial the TT genotype was significantly and independently associated with premature CAD [32]. Gardemann and colleagues [33] studied young individuals with high risk atherosclerotic profile and reported an association between 894G>T eNOS polymorphism and CAD in this cohort. To our knowledge, this study is one of the largest specifically designed study conducted in a general Caucasian population to test the possible association of this polymorphism with risk of AMI. Our results have not validated the association between G^894^→T polymorphism, in the eNOS gene and increased risk of AMI.

### Extent of CAD and in-hospital mortality in relation to eNOS genotypes

We have found no association of the studied genotypes with extent of CAD and in-hospital mortality. Gorchakova et al, recently showed that carriers of the mutated genotype have an increased risk of death or MI within one year after coronary artery stenting [34].

#### Study limitations

The main limitation of this study is the lack of data on eNOS activity that might have enabled us to establish correlations of the studied genotype with both the intermediate (NO levels) and final phenotype (AMI) of interest.

## Conclusion

In conclusion, this relatively large scale study, devalues the hypothesized association of the G^894^→T polymorphism in the eNOS gene with risk of AMI which had been verified in a series of relatively small association studies.

## Competing interests

The authors declare that they have no competing interests.

## Authors' contributions

GKA and EWH carried out the molecular genetic studies, participated in the sequence alignment and drafted the manuscript. DKG, SET and DCS carried out the immunoassays. SIZ and DJR participated in the sequence alignment, SGF, MNZ and EJG participated in the design of the study and performed the statistical analysis. ASM, PKT and CIS conceived of the study, and participated in its design and coordination and helped to draft the manuscript. All authors read and approved the final manuscript.

## Pre-publication history

The pre-publication history for this paper can be accessed here:


